# Effect of Milk Thistle, *Silybium marianum*, Extract on Toxicity, Development, Nutrition, and Enzyme Activities of the Small White Butterfly, *Pieris rapae*

**DOI:** 10.1673/031.013.14601

**Published:** 2013-12-07

**Authors:** Seyedeh M. Hasheminia, Jalal J. Sendi, Khalil T. Jahromi, Saeid Moharramipour

**Affiliations:** 1 Department of Entomology, Science and Research Branch, Islamic Azad University, Tehran, Iran; 2 Department of Plant Protection, Faculty of Agricultural Sciences, University of Guilan, Rasht, Iran; 3 Department of Plant Protection, College of Agriculture, University of Tehran, Karaj, Iran; 3 Department of Entomology, Tarbiat Modares University, Tehran, Iran

**Keywords:** methanolic extract, feeding indices, enzymatic compounds, biochemistry

## Abstract

The methanolic extract of milk thistle, *Silybium marianum* L. (Asterales: Asteraceae), was investigated for its effects on the mortality, growth, feeding indices, enzymatic activity, and levels of non-enzymatic molecules of the small white butterfly, *Pieris rapae* L. (Lepidoptera: Pieridae), a pest of cruciferous plants. Feeding indices including approximate digestibility (AD), efficiency of conversion of digested food (ECD), efficiency of conversion of ingested food (ECI), relative growth rate (RGR), and relative consumption rate (RCR) were measured. These indices were variously affected: the RGR, RCR, and AD decreased, but the ECD and ECI increased. The LC50 and LC25 values were estimated as 2.94% and 1.20%, respectively. At the lowest concentration of *S. marianum* extract (0.625%), the feeding deterrence index was 40.48%. The duration of the pupal stage and the rate of larval growth decreased. These changes may be due to alterations in metabolic activity, such as the increase in alkaline phosphatase activity, which is likely involved in detoxification. Additionally, the activities of alanine aminotransferase and aspartate aminotransferase, which are key components of amino acid catabolism, decreased. The amount of glucose (an energy source) and uric acid (the excreted end product) increased, while total protein (another energy source) and cholesterol decreased. These results indicate that this plant possesses potential secondary metabolites that may be useful for the future study of the control of insect pests.

## Introduction

Plant-derived extracts and phytochemicals have been intensively investigated for the past 30 years in an effort to develop alternatives to conventional insecticides with reduced health and environmental impacts. Synthetic insecticides can leave potentially toxic residues in food products and be deleterious to non-target organisms (Isman 2006).

Ferry et al. (2004) believed that crop losses due to pests may reach 10–20% annually. Synthetic chemical insecticides can have acute and chronic health effects on applicators, farmers, and even consumers; wildlife and beneficial insects, including pollinators, are also exposed. Insecticides also pollute underground water, endangering human health and the environment. The overuse of some chemical classes of insecticides has led to environmental problems (Isman 2006). These drawbacks associated with potent broadspectrum insecticides and their discontinued manufacture have spurred the search for biological pesticides with “softer” chemistries that are found in natural products. Among these natural products, botanical-based pesticides are chemicals from plant sources that may affect herbivores as toxins, growth regulators, repellents, deterrents, and compounds that affect digestibility (Koul et al. 2005). Plantbased bio-pesticides are generally thought to be more selective, be less harmful to nontarget organisms, degrade quickly, and be less phytotoxic (Kim et al. 2003). Therefore, botanical insecticides may be effective for managing the small white butterfly, *Pieris rapae* L. (Lepidoptera: Pieridae), which is an important pest that affects cruciferous crops worldwide. A single female lays 300 eggs on average, but clutches may include close to 1000 eggs. Each young larva occupies an outer leaf of a cabbage for feeding. Older larvae move onto inner cabbage leaves and into cabbage heads by eating outer leaves of the cabbage head and gnawing into it. Plants soiled with excrement rot easily (Jogar et al. 2009). Hasheminia et al. (2011) showed that *Artemisia annua* L. and *Achillea mellifolium* L. crude leaf extracts affected the longevity, feeding efficiency, and chemical activities of *P. rapae.*

*Silybum marianum* (L.) Gaertn (Asterales: Asteraceae) is an important medicinal plant commonly known as milk-thistle, or St. Mary's thistle. The plant and its extracts are reported to possess hepatoprotective, antioxidant, anticancer, anti-inflammatory, and antidiabetic properties. It contains the flavonolignan Silymarin, which is an important bioactive compound with anticancer, antiinflammatory, antioxidant, and immunomodulatory effects (Balian et al. 2006).

Compounds derived from plant sources may influence the physiology of target insects in many ways. This study reports the biochemical effects of milk-thistle extract on *P. rapae.* We studied aspartate amino transferase (AST, EC 2.6.1.1) and alanine amino transferase (ALT, EC 2.6.1.2) because they are considered important links between carbohydrate and protein metabolism. These enzymes' activities change upon various physiological and pathological conditions (Etebari et al. 2005). Alkaline phosphatase (EC 3.1.3.1) is a hydrolyzing enzyme responsible for removing phosphate groups from many types of molecules including nucleotides, proteins, and alkaloids (Miao 2002).

Alpha amylase is an enzyme important in the metabolism of starch and other carbohydrates. Uric acid is the excreted end product in insects, and its amount correlates with the amount of protein in the insect's body. The cholesterol in phytophagous insects is made through plant sterols, and insects convert them to ecdysteroids for ecdysis (Chapman 1998). Several insect activities depend on carbohydrate metabolism. The amount of glucose indicates the availability of this sugar for carbohydrate metabolism in insect cells (Satake et al. 2000).

In this study, we describe the toxicology and other biological properties of milk-thistle on a very common but important lepidopterous pest, *P. rapae.*

## Materials and Methods

### Mass culture of P. *rapae*

*P. rapae* adults were collected in cabbage fields near Mehrshahr, Karaj and were released into a net cage (3 × 1.5 × 2.5 m). Seven cabbage plants were also enclosed in the cage on which the butterflies could lay eggs. Weeds such as *Malva sylvestris* and *Convolvulus arvensis* were provided so that the adults could feed upon their nectar. Newly hatched larvae were transferred to 15 × 30 cm plastic jars with cabbage foliage and grown in a growth chamber with a 16:8 L:D photoperiod at 25 ± I^o^ C and 65 ± 5% RH.

### Methanolic extract preparation

Milk-thistle *S. marianum* seeds were obtained from the Institute of Medicinal Plants in Karaj, Iran in July, 2009. They were washed with distilled water and dried at room temperature in the shade. The seeds were pulverized into a powder by electric crushing. Methanolic extraction was carried out according to the procedure described by Warthen et al. (1984). Briefly, 30 g of seed powder was stirred with 300 mL of 85% methanol in a flask for 1 hour. The methanolic solution was incubated for 48 hours at 4° C and then stirred for an additional hour and filtered through Whatman No. 4 fllter paper. The solvent was removed by vacuum in a rotary evaporator, and the residue was dissolved in 10 mL of methanol and used as a starting stock solution. Further dilutions with methanol were used to prepare solutions at various concentrations.

### Bioassays and treatment

**Toxicity tests.** Five concentrations of *S. marianum* extract, 0.625, 1.25, 2.5, 5, and 10%, and a control methanol treatment were used for toxicity tests and the determination of the LC50 and LC25 values. Each bioassay was performed with 3^rd^ instar *P. rapae* larvae, and 30 larvae per concentration were used for all the experiments. The experiments were replicated three times. Mortality was recorded at 48 hours, and the LC_50_ and LC_25_ values were calculated using probit analysis and POLO-PC software (Leora 1987).

**Deterrence tests.** Two sections of cabbage leaf of equal size were cut and dipped in the desired concentration of *S. marianum* extract for 30 seconds. Then they were dried at room temperature. After 4 hours of starvation, 3^rd^ instar larvae were placed onto control or treated pieces of leaf placed in 15 × 30 cm plastic jars. Leaf consumption was recorded using a digitizing leaf area meter (ADC BioScientific Ltd., http://www.adc.co.uk) after 24 hours. The index of feeding deterrence was calculated as (C - T)/(C + T) × 100, where C is the consumption of the control leaf and T is the consumption of the treated leaf. Each experiment was repeated three times with 10 larvae each time.

**Feeding, growth, and dietary utilization of** **plant extracts.** The duration of the larval, pupal, and adult stages and the numbers of deformed insects after treatment with different concentrations of the methanolic extract were evaluated. Several concentrations were prepared (0.625, 1.25, 2.5, 5, and 10%), and the cut leaves were dipped in the desired concentration for 30 seconds and dried at room temperature. Control leaves were dipped in methanol and dried as described above. Control and treated leaves were placed in 15 × 30 cm plastic jars, and then 3^rd^ instars starved for 4 hours were introduced. Control and treatment larvae were fed fresh leaves and maintained in a growth chamber with a 16:8 L:D photoperiod at 25 ± 1° C and 65 ± 5% RH. The durations of the life stages were recorded.

Short-term feeding trials with five concentrations of *S. marianum* extract (0.625, 1.25, 2.5, 5, and 10%) were conducted to evaluate the effects on the growth rates, food consumption rates, and food processing efficiencies of the larvae. The controls were treated with methanol alone. Ten newly hatched larvae were grown on cabbage foliage in a growth chamber with a 16:8 L:D photoperiod at 25 ± 1° C and 65 ± 5% RH. Each experiment was repeated three times. Each assay consisted of a newly molted and weighed larva placed into a rearing plastic jar (15 × 30 cm) containing a leaf from a cabbage plant. The leaves were changed every 1–2 days or as necessary during the bioassay. Upon molting to 4th instars, the larvae were oven-dried at 60° C for one week and re-weighed. The nutritional indices were calculated to evaluate insect growth, consumption, and food utilization efficiency. These indices were calculated from standard formulas (Hare 1998): approximate digestibility (AD) (Ingestion - Feces)/Ingestion), efficiency of conversion of digested food (ECD) (Biomass gained/(Ingestion - Feces), and efficiency of conversion of ingested food (ECI) (Biomass gained/Ingestion). Initial rather than average weights of the larvae were used to calculate the relative growth rate (RGR) and the relative consumption rate (RCR) (Farrer et al. 1989). The oven-dried weights of the feces and the remaining leaf fragments were obtained by drying them at 105° C for 24 hours. The dry material was allowed to cool in desiccators before weighing. To obtain the dry weights of the leaves before feeding, fresh leaves were used to obtain a correlation between dry weight and fresh weight. The best predictor of dry leaf weight (Y) was: Y = 0.4198X + 0.0003, where X is fresh leaf weight.

### Biochemical analysis

About 25 freshly enclosed 3^rd^ instar *P. rapae* larvae were killed by freezing, and whole body extracts were obtained 24 hours after treatment. The samples were diluted with phosphate buffer (1:1, w/v) and centrifuged for 10 min at 14000 rpm. The supernatants were transferred to new tubes and stored at -30° C until use. Each biochemical analysis was repeated three times. Alanine aminotransferase (ALT) and aspartate aminotransferase (AST) activities were measured using Thomas' procedure (Thomas 1998). Alpha amylase was measured using the Henry and Chiamari (1960) method. Briefly, 2-choloro-4-nitrophenyl D-maltotrioside, in which 2-chloro-4-nitrophenol has been bound to maltoriose, is hydrolyzed by alpha-amylase, and the enzymatic activity is determined at 405 nm. Alkaline phosphatase (EC 3.1.3.1) activity was measured as described by Mihara et al. (1988). The substrate was incubated with tissue extract for 30 min, and the reaction was stopped by adding alkali. The spectral absorbance of p-nitrophenolate was read at 310 nm. Protein was measured based on the Biuret method as described by Reinhold (1953) using a protein assay kit (Pars Azmon Co, Iran, http://parsazmun.ir) and measuring the absorbance at 540 nm. Glucose was analyzed as described by Siegert (1987), and total cholesterol was measured as described by Richmonds (1973) by hydrolyzing cholesterol esters with cholesterol oxidase, cholesterol esterase, and peroxidase. Uric acid content was determined using uricase as described by Valovage and Brooks (1979) at a wavelength of 500 nm.

### Statistical analysis

For determination of the mortality rate and the lethal concentration, probit analysis was conducted using POLO-PC (Le Ora 1987). A factorial experimental design was laid out in a completely randomized fashion with three replicates. All data were subjected to analysis of variance. Duncan's multiple range tests were used for separating treatment means at *p =* 0.05 (SAS 1997). Differences between samples were considered statistically significant at p < 0.05.

## Results

**Figure 1.**
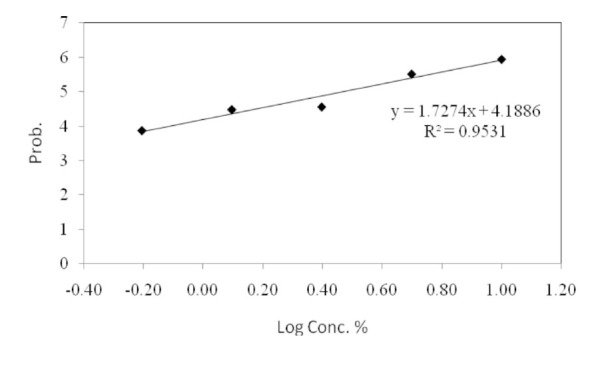
Dose response of *Pieris rapae* L. 3^rd^ instar larvae to *Silybum marianum* extract. High quality figures are available online.

The LC50 and LC25 values, the confidence limit (95%), and the regression slope at 48 hours of exposure to plant extracts are depicted in Table 1 and Figure 1. The** LC50** and **LC25** values for *S. marianum* were 2.94 and 1.20%, respectively. The mean deterrency of 3rd instar *P. rapae* larvae to leaves treated with the lowest dose (0.625%) of methanol extract of *S. marianum* was 40.479 ± 5.23% compared to the control feeding with methanol-treated leaves. The duration of the larval and pupal periods were slightly reduced upon treatment with various concentrations of *S. marianum* (Table 2). However, the percentage of adult emergence was significantly decreased upon *S. marianum* treatment. At 2.5%, no adult emergence occurred. The emerged adults exhibited pronounced deformities in the wings (adultoids). The low dose of 0.625% of the *S. marianum* extract resulted in a significant increase in ECI and ECD, a significant decrease in RGR and RCR, and no change in AD compared with the controls (Table 3). The effects of the 0.625% dose milk-thistle extract on enzymatic activity and levels of several molecules are shown in Table 4. The amounts of glucose and uric acid significantly increased, while the amount of cholesterol significantly decreased. a-amylase, aspartate aminotransferase, and alanine aminotransferase activity significantly decreased after *S. marianum* treatment compared to the control, while alkaline phosphatase activity was significantly increased.

**Table 1.**
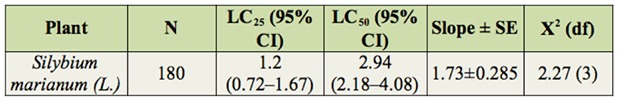
Dose-response parameters of 3^rd^ instar larvae of Pieris rapae L. 48 hours after exposure to *Silybum marianum* extract.

**Table 2.**
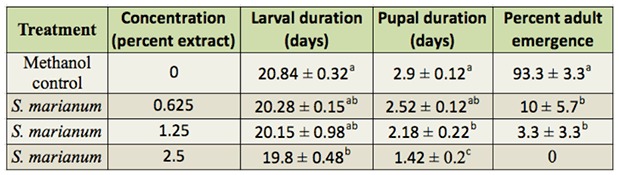
The effect of *Silybum marianum* extract on the durations of the life stages of *Pieris rapae* L. Within columns, means (SE) followed by the same letter do not differ significantly *(p* > 0.05).

**Table 3.**
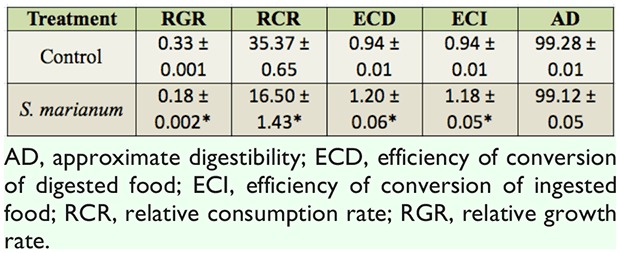
Nutritional indices of 3^rd^ instar *Pieris rapae* L. larvae after treatment with 0.625% *Silybum marianum.* An asterisk indicates a significant difference relative to the control treatment *(p* > 0.05).

**Table 4.**
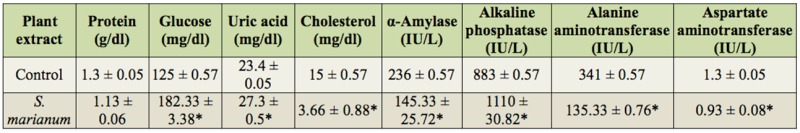
Effects of *Silybum marianum* extract on biochemical compounds of *Pieris rapae* L. 3^rd^ instar larvae. An asterisk indi:ates a significant difference relative to the control treatment *(p >* 0.05).

## Discussion

Secondary metabolites produced by plants play a major role in insect defenses against herbivores because of their toxicity, antifeedant activity, and effects on growth and development (Isman 2006).

Our results showed that treatment with *S. marianum* extracts was toxic at high doses, and at low doses it acted as an antifeedant, interfering with key metabolic pathways.

The toxic effects of *S. marianum* are clearly indicated by the low LC_50_ values after 48 hours of exposure. Earlier reports on Asteraceae clearly depict the potential of the species of this plant family to have toxic properties against pests (Jalali et al. 2002, 2005; Tripathi et al. 2003; Negahban et al. 2007; Shekari et al. 2008). Hence, this finding is consistent with earlier reports.

The deterrency index obtained in the present study clearly shows the optimal effect expected upon treatment with *S. marianum* extract. Deterrency could be attributed to the effects of the chemicals present in the extract on chemoreceptors (Schoonhoven and Fu-Shun 1989; Gershenzon and Croteau 1992). The chemicals may block the stimulant effects of glucose, sucrose, and inositol on chemore-ceptors (Zapata et al. 2009).

Larval and pupal duration, though expected to increase based on other reports (Senthil Na-than et al. 2005; Senthil Nathan and Sehoon 2006), did not change upon low doses of plant extract and decreased upon higher doses. Disruption of the endocrine system could explain the non-emergent and deformed adults (adultoids).

The RCR was significantly reduced by the extracts compared to the control, which is consistent with the deterrency index. There was no change in AD, but both the efficiency of the conversion of digested food and the efficiency of the conversion of ingested food were increased by *S. marianum* extract. The unchanged AD is consistent with the result obtained for *Cnapholocrocis medinalis* Guenée upon treatment with *Melia azaderach* L. (Senthil Nathan 2006). The author of that study speculated that AD could not be main-tained due to a reduced RGR. We also report a reduced RGR upon treatment with *S. marianum* extract in the present insect, which is consistent with the effect of the plant product fraxinellone on *Ostrinia fiirnacalis* Guenee (Liu et al. 2008). In that study, the authors related the reduction in growth to post-ingestive toxicity of the compound and found a reduced efficiency of the conversion of ingested food. However, we found a slight increase in both the efficiency of the conversion of ingested food and the efficiency of the conversion of digested food, suggesting that this phenomenon is complicated. Various factors affect the physiological condition of an organism, in-cluding chemical stress. The present result clearly indicates changes brought about by *S. marianum* extract in *P. rapae.*

Aminotransferases are enzymes that catalyze a reaction between an amino acid and a keto acid. This reaction, called transamination, involves removing the amino group from the amino acid, leaving behind a keto acid and converting it into an amino acid.

These enzymes serve as strategic links between carbohydrate and protein metabolism that are known to be altered during various physiological processes (Etebari et al. 2005). Our study showed sharp decreases in alanine aminotrasferase and aspartate aminotransferase upon treatment with plant extracts, which is similar to the results obtained by Mandai (1983) where allatectomy led to reductions in aspartate aminotransferase and alanine aminotransferase activity in female and male *Lohita grandis* Gray. Treatment with juvenoid reversed the effect of allactectomy. The endocrine disruption indicated by an increased larval duration upon treatment with the plant extracts could be due to reduced aspartate aminotransferase and alanine aminotransferase activity.

Alkaline phosphatase (EC 3.1.3.1) is a hydrolytic enzyme responsible for removing phosphate groups from many types of molecules including nucleotides, proteins, and alkaloids. In most studies testing the effect of toxic chemicals, alkaline phosphatase is decreased (Yoshitake et al. 1966; Eguchi and Iwamato 1975), as it is in studies testing plant extracts (Senthil Nathan et al. 2006; Shekari et al. 2008). Our study shows that alkaline phosphatase is increased by the treatment with *S. marianum* extracts, which suggests a possible involvement in detoxification.

α-amylase is an enzyme that hydrolyzes the alpha bond of large alpha-linked polysaccharides such as starch and glycogen, yielding glucose and maltose. The present study clearly depicts its lower activity following treatment with *S. marianum* extract, which is consistent with other reports on various chemical treatments (Saleem and Shakori 1987; Lee et al. 1994; Shekari et al. 2008; Zibaee et al. 2008). Glucose concentrations increased following treatment with plant extracts. Earlier studies have also shown glucose increases after treatment with chemicals like pyriproxyfen, fenitrothion, ethion, and azadirachtin (Mukherjee et al. 1993; Nath 2003; Etebari et al. 2005). The increase in glucose may be due to enhanced trehalase activity because it was reported that trehalase activity increased in the midgut of silkworms treated with insecticides (Vyjayanthi and Subramanyam 2002).

A sharp decrease in cholesterol after treatment with plant extract was also observed. Decreases in compounds like cholesterol could be attributed to physiological stress or interruptions in the absorption system (Etebari et al. 2005). Uric acid is the excreted end product in insects, and its amount correlates with the amount of protein in the insect's body. The increase in uric acid could be due to altered metabolic pathways after treatment that prevented the natural excretion of uric acid (Etebari and Matindoost 2004).

In conclusion, the present results indicate toxic, deterrent, and feeding inhibitory effects of *S. marianum* extracts on *P. rapae.* Moreover, the secondary chemicals present in the extract affect enzymatic activities and the levels of non-enzymatic molecules in this insect.
